# Recent Advancements in Sensor Technologies for Healthcare and Biomedical Applications

**DOI:** 10.3390/s23063218

**Published:** 2023-03-17

**Authors:** Wenfeng Zheng, Yichao Yang, Chao Liu, Wenshuo Zhou

**Affiliations:** 1 School of Automation, University of Electronic Science and Technology of China, Chengdu 610054, China; 2 Department of Pharmaceutical Sciences, School of Pharmacy, Bouve College of Health Sciences, Northeastern University, 140 The Fenway, Boston, MA 02115, USA; yicy07@163.com; 3 LIRMM, UMR 5506, University of Montpellier-CNRS, 34095 Montpellier, France; liu@lirmm.fr; 4 Lab of Immunoregulation, Division of Viral Products, Office of Vaccines Research and Review, Center for Biologics, FDA, 10903 New Hampshire Ave., Silver Spring, MD 20993, USA; zhouwenshuobiology@gmail.com

Biomedical sensors are the key units of medical and healthcare systems. The development focus of this topic is to use new technology and advanced functional biocompatible materials to design miniature, intelligent, reliable, multifunctional, low-cost, and efficient sensors. The last two decades have seen unprecedented growth in the employment of advanced sensors, which enable the detection of critical biomarkers for the early diagnosis of human diseases and the monitoring of human physiological signals for assessments in healthcare and biomedical applications. This rapid progress in both sensor technology development and its applications is mainly due to the quickly advancing development of micro/nanofabrication, manufacturing techniques, and advanced materials, as well as the increasing demand for the development of fast, simple, and sensitive measurement techniques that are capable of accurately and reliably monitoring biological samples in real time. The development of biomedical sensors is driven by the requirements of the medical field. The screening and continuous monitoring of patients with sensors has become increasingly important. A huge growth in the demand for home care will certainly promote the development of disposable sensors or telemedicine. This also puts forward requirements for future medical sensors.

This Special Issue aims to provide an overview of recent advancements in the area of sensing technologies, including of sensors and platforms with a focus on functional materials, novel sensing mechanisms, design principles, fabrication and characterization techniques, performance optimization methods, multifunctional and multiplex sensing platforms, and system integration strategies, which play crucial roles in many applications.

Gökhan Güney et al. [1] used MediaPipe artificial intelligence (AI)-based hand-tracking technologies to quantitatively assess the hand movements of patients that were suffering from Parkinson’s Disease (PD). First, they investigated the frequency and amplitude relationship between the video and accelerometer data. Then, they focused on quantifying the effects of taking standard oral treatments. Their work achieved an automatic estimation of the movement frequency and tremor frequency with a low error rate, and this appears to be the first paper that has presented an automated tremor analysis before/after the use of medication for PD, and, in particular, the first to use high-frame-rate video data.

Athanasios Tsanas et al. [2] proposed a new acceleration summary measure, the Rate of Change Acceleration Movement (ROCAM), and compared its performance against three established approaches, summarizing the three-dimensional acceleration data to replicate the minute-by-minute labels. Moreover, they compared findings where the acceleration data was sampled at 10, 25, 50, and 100 Hz. Collectively, this study contributed new insights into the analysis of wrist-worn actigraphy data in three areas, and provided insights into facilitating the deployment of large-scale, longitudinal actigraphy data processing for 24 h physical activity and sleep profile assessments.

Negin Foroughimehr et al. [3] demonstrated the accuracy variation of the finite-difference time-domain (FDTD) computational simulation system when different meshing sizes were used, by using the interaction of the critically sensitive human cornea with EM within the 30 to 100 GHz range. Different approaches to the base cell size specifications were compared. The accuracy of the computation was determined by applying planar sensors that showed the detail of the electric field distribution, as well as the absolute values of the electric field that were collected by point sensors. The results of the presented simulations suggested that using an adaptive cell size specification provided fewer radiation artifacts, resulting in more accurate computational simulations. Furthermore, they found that the adaptive cell size setup radically increased the required computation time compared with the manual specification of the cell sizes.

David Burns et al. [4] presented an approach to personalized activity recognition that was based on deep feature representation derived from a convolutional neural network (CNN). They experimented with both categorical cross-entropy loss and triplet loss for training, and described a novel loss function based on the subject triplets. These results showed that the personalized algorithms they presented were more robust to inter-subject variability in inertial time series datasets. They significantly outperformed impersonal approaches in more challenging classification tasks, where there exists a high degree of similarity between classes.

In the study by Zixi Gu et al. [5], a real-time muscular activity measurement system using non-contact sensors was developed. The system used two inertial measurement unit (IMU) sensors to collect the motion data that was produced during normal walking, in order to estimate the knee extensor activity. An artificial neural network was used in the estimation model training. An evaluation experiment was also conducted to validate the estimation algorithm. The muscle activity estimation results, which were estimated by the proposed algorithm after its optimization, showed a relatively high estimation accuracy, with a correlation efficient of R^2^ = 0.48 and a standard deviation STD = 0.10, with a total system average delay of 192 ms. Compared with the previous study, this newly proposed system presented a higher accuracy and was more suitable for real-time leg muscle activity estimation during walking.

Chisaki Miura et al. [6] aimed to develop a virtual caregiver system that retrieved expressions of mental and physical health states through a human–computer interaction in the form of dialogue. The purpose of this paper was to implement and evaluate a virtual caregiver system using a mobile chatbot. Unlike the conventional health monitoring approach, their key idea was to integrate a rule-based virtual caregiver system (called a “Mind Monitoring” service) containing physical, mental, and social questionnaires into the mobile chat application. According to the main results, its effects were significantly improved by the proposed method, including an 80% response rate, an accurate reflection of real lives in the responses, and a high usefulness of the feedback messages with regard to the software quality requirements and evaluation.

Robert Karpiński et al. [7] proposed a method for processing acoustic signals and selecting optimal signal measures that used the neighborhood component analysis (NCA) algorithm. Their obtained results confirmed the thesis that an inexpensive, noninvasive, and, most importantly, effective diagnosis of damage to the articular cartilage that covers the articular surfaces of the patellofemoral joint, based on generated vibroacoustic signals, was possible. This confirmed the validity of the assumptions that were made and the usefulness of the proposed method that was created based on statistical parameters and machine learning.

Aarón Cuevas-López et al. [8] presented a digital compression algorithm that was capable of reducing electrophysiological data to less than 65.5% of its original size, without distorting the signals. This algorithm could compress neural data to nearly half its original size in a lossless manner, without adding any distortion. The power required by the algorithm itself was less than 3 mW, which was negligible compared to the power that was saved by reducing the transmission bandwidth requirements. These developments could be used to create a variety of wired and wireless neural electrophysiology acquisition systems with low power and space requirements, without the need for complex or expensive specialized hardware.

Sheng-Wei Pan et al. [9] proposed a characterization method that used attenuated total reflection-Fourier transform infrared spectroscopy (ATR-FTIR) spectra to evaluate urine samples, and assessed the correlation between the ATR-FTIR patterns, urinary tract infection (UTI) diagnoses, and causative pathogens. Their results indicated that the relative ratios between the different area zones of vibration, as well as the multivariate analysis, could be used as a clue to discriminate between UTIs and non-UTIs, as well as the different causative pathogens of UTIs. Their findings suggested that this calculation method using ATR-FTIR may provide clues to detect UTIs and other diseases in the future.

Lalita Chopra et al. [10] prepared multifunctional binary graft copolymeric matrices of chitosan with monomer acrylic acid (AA) and various comonomers acrylamides (AAm) and acrylonitriles (AN), by performing free radical graft copolymerization in the presence of an initiator potassium persulfate (KPS). These binary grafts showed significantly better-controlled drug diffusion than the unmodified backbone, and produced superior results compared to the chitosan. The graft copolymer Ch-graft-poly (AA-cop-AAm) provided superior results with regard to sustainable drug release, as well as for the metal ion uptake. The study explored the potential of chitosan-based materials in the industry, as well in the biomedical field.

Wenfeng Zheng et al. [11] proposed a low-dose computed tomography (CT) image post-processing method based on learning sparse transform. Their image post-processing method did not need to obtain the real projection data, and the method reduced the research threshold, could realize offline processing, and was easy to use. In this paper, two different learned sparse transformations were used. The first covered more organizational information about the scanned object, and the other could cover more noise artifacts. Both methods could improve the ability to learn sparse transformations, in order to express various image information. These experimental results showed that the algorithm was effective.

Robert Karpiński et al. [12] aimed to establish diagnostic accuracy and to identify the most accurate signal processing method for the detection of osteoarthritis (OA) in knee joints. An analysis of their results showed that vibroarthrography can be an effective, low-cost, and accurate diagnostic modality for the evaluation of cartilage damage in tibiofemoral joints, and that it can be implemented in daily orthopedic practice. A neighborhood component analysis (NCA) algorithm was used for the detection of signals and the optimization of the quantity of the input data, aiding in the maximization of the classification accuracy in a shorter calculation time.

Dan Wang et al. [13] explored approaches to the application of mechanical stimuli to different cell types using kidney-on-a-chip models, and examined how these systems are used to study kidney physiology, to model disease, and to screen for drug toxicity. They further discussed sensor integration into kidney-on-a-chip models for the monitoring of cellular responses to mechanical or other pathological stimuli. They discussed the advantages, limitations, and challenges associated with incorporating these mechanical stimuli into kidney-on-a-chip models for a variety of applications. Overall, this review aimed to highlight the importance of mechanical stimuli and sensor integration in the design and implementation of kidney-on-a-chip devices.

Pablo Campo-Prieto et al. [14] explored whether a commercial wearable head-mounted display (HMD) and a selected virtual reality (VR) exergame could be suitable for people with mild–moderate Parkinson’s Disease (PD). In all, 32 patients (78.1% men; 71.50 ± 11.80 years) were a part of the study. Its outcomes supported the feasibility of a boxing exergame combined with a wearable commercial HMD as a suitable physical activity for PD, and reinforced its applicability in different environments due to its safety, usability, low cost, and small size.

We would like to express our profound appreciation to the authors and reviewers who made this Special Issue possible. In the time it took to complete this Special Issue, our reviewers and authors contributed tremendous efforts to improve the paper’s quality and thus guarantee the high standard of this Special Issue.
